#  Asimetrías entre la coordinación nacional y la autonomía de los gobiernos estatales en la adopción de tecnologías sanitarias frente a la COVID-19 en Argentina 

**DOI:** 10.1590/0102-311XES117923

**Published:** 2024-04-29

**Authors:** Santiago Hasdeu, Anabel Beliera, Jorgelina Alvarez, Julian Sanchez-Viamonte

**Affiliations:** 1 Universidad Nacional del Comahue, Neuquén, Argentina.; 2 RedARETS, Neuquén, Argentina.; 3 Universidad Nacional de Cuyo, Mendoza, Argentina.; 4 Universidad Nacional de Mar del Plata, La Plata, Argentina.

**Keywords:** Política Pública, Federalismo, Toma de Decisiones en la Organización, Infecciones por Coronavirus, Evaluación de la Tecnología Biomédica, Public Policy, Federalism, Organizational Decision Making, Coronavirus Infections, Biomedical Technology Assessment, Política Pública, Federalismo, Tomada de Decisões Gerenciais, Infecções por Coronavirus, Avaliação da Tecnologia Biomédica

## Abstract

En pandemia, en Argentina y en otros países se observó variabilidad en las políticas públicas implementadas sobre tecnologías sanitarias para prevención y tratamiento de la COVID-19. El objetivo fue analizar cómo se procesaron en Argentina los movimientos de coordinación vs. cooperación, y de autonomía vs. reparto de autoridad entre entidades, explorando asimetrías entre diferentes entidades en la implementación de políticas públicas sobre tecnologías sanitarias en pandemia y las influencias de otros actores. Se realizó una revisión documental del período 2020-2021 (informes técnicos publicados por la Organización Mundial de la Salud, organismos nacionales y sociedades científicas, leyes, fallos judiciales, prensa, encuestas y entrevistas en profundidad a miembros de los Ministerios de Salud de Argentina). Se indagó sobre procesos y resultados de la toma de decisiones en los Ministerios de Salud, mapeando la cobertura y recomendación de cada tecnología y el partido político provincial gobernante. Hubo heterogeneidad en resultados y procesos entre los Ministerios, y disputas en el interior de los mismos. La adherencia a recomendaciones oficiales fue baja, influyendo distintos criterios técnico-políticos (relaciones de poder, presión social, de los medios, académicos, poder Judicial y Legislativo). En algunos casos se observó una fuerte tensión entre oficialismo y oposición al partido gobernante a partir de la discusión sobre tecnologías. Cada provincia argentina definió sus políticas sobre tecnologías sanitarias para COVID-19 con autonomía, y la toma de decisiones en la administración pública en pandemia fue desordenada, compleja y no lineal.

## Abstract (en)

Argentina, as other countries, showed several public policies related to the health technologies implemented to fight and treat the COVID-19 pandemic. This study sought to analyze how articulation vs. cooperation and autonomy vs. division of powers between entities occurred in Argentina, exploring asymmetries between several entities in implementing public policies related to health technologies during the pandemic and the influences of other actors. For this, a documentary research was carried out related to 2020-2021 (technical reports published by the World Health Organization, national agencies and scientific societies, laws, court decisions, press, and research and in-depth interviews with members of the Argentine Ministries of Health). The processes and results of decision-making in the Ministries of Health were analyzed, outlining the coverage and orientations of each technology and the political party in power in the province. This study found heterogeneous results and processes between Ministries and disputes within them. It also observed the poor adherence to official guidelines due to technical-political criteria (power relations, social, media, academic, judiciary, and legislative pressure). Some cases showed a strong tension between the government and its opposition over the discussion of technologies. Each province in Argentina has autonomously defined its policies on health technologies for COVID-19, and decision-making in public administration was disorderly, complex, and non-linear during the pandemic.

## Resumo

Na Argentina, assim como em outros países, houve uma variabilidade de políticas públicas relacionadas às tecnologias de saúde implementadas no combate e tratamento da COVID-19 durante a pandemia. Este estudo buscou analisar como ocorreram a articulação *vs.* cooperação, e a autonomia *vs.* divisão de poderes entre as entidades na Argentina, explorando assimetrias entre diferentes entidades na implementação de políticas públicas relacionadas a tecnologias de saúde na pandemia, bem como as influências de outros atores. Para tanto, realizou-se uma pesquisa documental para o período de 2020-2021 (relatórios técnicos publicados pela Organização Mundial da Saúde, agências nacionais e sociedades científicas, leis, decisões judiciais, imprensa, pesquisas e entrevistas em profundidade com membros dos Ministérios da Saúde da Argentina). Os processos e os resultados da tomada de decisão nos Ministérios da Saúde foram analisados, traçando a cobertura e orientações de cada tecnologia e o partido político no poder na província. Observou-se uma heterogeneidade nos resultados e processos entre os Ministérios, e disputas em seu interior. Houve uma baixa adesão às orientações oficiais, influenciada por diferentes critérios técnico-políticos (relações de poder, pressão social, midiática, acadêmica, do poder Judiciário e do Legislativo). Em alguns casos, observou-se uma forte tensão entre governo e oposição a partir da discussão das tecnologias. Cada província da Argentina definiu suas políticas sobre tecnologias de saúde para a COVID-19 de forma autônoma, e a tomada de decisões na administração pública foi desordenada, complexa e não linear durante a pandemia.

## Introducción

El proceso de toma de decisiones en la administración pública es investigado desde diferentes miradas y disciplinas. Desde los años 1990 se enfatizó en el diseño de políticas basadas en evidencias, pero esta no es la única base sobre la cual se diseñan políticas públicas ^1^. Se ha mencionado la influencia que tienen distintos criterios técnicos y políticos, la experiencia del decisor, su perfil, sus valores, las características del equipo de asesores, las relaciones de poder dentro y fuera de la administración, medios de comunicación, presión social y el contexto político y económico ^1^^,^^2^.

El sistema de salud argentino se encuentra fragmentado en diversos subsectores ^3^, con gran heterogeneidad en términos de población a cargo, indicadores epidemiológicos, financiamiento, y reglas administrativas bajo las cuales se rigen ^4^. La Comisión Nacional de Evaluación de Tecnologías Sanitarias de Argentina (CONETEC), emitía recomendaciones no vinculantes hasta 2023 ^5^, por lo que cada subsector de salud tomaba sus propias decisiones sobre tecnologías sanitarias. Se describió escasa vinculación entre los equipos técnicos de evaluación de tecnologías sanitarias (ETS) y los tomadores de decisión en Argentina, lo que podría relacionarse con su limitado impacto en las políticas públicas ^6^^,^^7^.

Las políticas públicas tienen lugar en un sistema complejo, que, aun observado y analizado, no termina de comprenderse con claridad, por lo que fue denominado “la caja negra gubernamental” ^8^. Se propuso matizar la racionalidad del decisor (racionalidad limitada) ^9^, y entender el comportamiento organizacional como un juego de poder, en el que actores influyentes, internos y externos, buscan controlar las decisiones y acciones, formando coaliciones que buscan ganar poder sobre la organización ^2^. Laswell ^10^ (p. 14) inicia el estudio de las políticas públicas (policies), definiéndolas como “*disciplinas que se ocupan de explicar los procesos de elaboración y ejecución de las políticas para resolver problemas públicos*”. Los procesos sociales sobre los que las políticas públicas intentan influir son conocidos parcialmente, la información sobre la que se basa la definición de los problemas y la elección de las alternativas es imperfecta, y los condicionantes políticos obligan a aplicar soluciones de compromiso que minimicen los conflictos de intereses; los conocimientos y las habilidades de los actores no son siempre las adecuadas para asumir los desafíos de implementación, y por último, las políticas producen, a menudo, impactos imprevistos ^11^. La formulación de políticas es una actividad sociocultural regida por leyes, pero inmersa en los procesos sociales cotidianos, en los “mundos de sentido” humanistas, sus protocolos lingüísticos y prácticas culturales ^12^. Por ende, un análisis meramente normativo o institucional presenta limitaciones, y se vuelve importante analizar las tramas de actores que se encuentran en estos procesos. El análisis de políticas públicas implica dar sentido al conocimiento tácito, a las múltiples interpretaciones, y a las definiciones en conflicto que las políticas tienen para los actores situados en lugares diferentes ^13^.

Uno de los indicadores más utilizados para evaluar la performance sanitaria ante la pandemia es la estimación de exceso de mortalidad. La Organización Mundial de la Salud (OMS) sitúa a Brasil entre los 20 países con mayor exceso de mortalidad por COVID-19, estimándola entre 20% y 30% ^14^, mientras para Argentina la estima entre 10% y 20%. La OMS reconoce diferencias subnacionales en estos y otros países de la región ^14^^,^^15^. Hasta julio 2023 las muertes por millón de habitantes por COVID-19 fueron 2.873,88 en Argentina, y 3.275,94 en Brasil ^16^.

En pandemia, Latinoamérica fue escenario de utilización masiva de medicamentos no recomendados por la OMS ^17^. En algunos casos esto fue promovido como política pública por los Estados nacionales, como en Perú y Bolivia, o subnacionales como el Municipio de Natal (Brasil), o la Provincia de Tucumán (Argentina), que incorporaron la ivermectina en sus guías terapéuticas ^17^^,^^18^. El denominado “kit COVID” (incluía ivermectina, hidroxicloroquina y azitromicina) fue entregado por la administración pública en diversos estados de Brasil. El gasto del gobierno de Bolsonaro en estos medicamentos no recomendados por la OMS fue millonario ^19^. Las ventas de ivermectina se incrementaron en forma masiva durante la pandemia en Brasil, objetivándose un incremento en los reportes de eventos adversos ^20^. Una revisión preliminar de documentos oficiales de los Ministerios de Salud de la Nación y de las provincias argentinas muestran políticas disímiles sobre ivermectina, plasma de convaleciente, suero equino, e ibuprofeno inhalado ^21^^,^^22^^,^^23^.

La Constitución Argentina tiene una división de poderes horizontal (Poderes Ejecutivo, Legislativo, Judicial), y una división vertical de poderes, porque se trata de un país federal con Nación, provincias y municipios ^24^. El federalismo argentino es cooperativo, habilitando competencias concurrentes entre Estado nacional y provincias ^25^. Habilita que distintos niveles de gobierno participen de manera conjunta y posean atribuciones para intervenir sobre las mismas temáticas por medio de políticas públicas con grados diversos de coordinación. El debate sobre la autonomía de las entidades subnacionales latinoamericanas enfatiza la naturaleza política de las acciones de los gobiernos estatales en un escenario en el que algunos autores consideran que dominan el patrimonialismo y el clientelismo en el sistema de toma de decisiones ^26^, mientras otros encuentran márgenes de acción de protección de las comunidades cuando las políticas públicas emanadas del Ejecutivo Nacional fueran perjudiciales ^27^. En este sentido, no debe confundirse federalismo con descentralización, pues esta última no es ni una condición necesaria ni suficiente para el primero ^28^. El federalismo trata la organización política territorial del poder mediante una matriz compartida de soberanía, no piramidal, manteniendo la estructura nacional ^29^. En esta forma de organización del poder del Estado, el nivel de gobierno central, de carácter nacional, y el descentralizado, constituido por gobiernos subnacionales, tienen ambos autonomía territorial, y los representantes tienen poderes exclusivos, y poderes en competencia sobre el mismo territorio y población ^30^. Más allá del análisis de las normativas que regulan la descentralización, es importante analizar los ejes: fiscal, administrativo y político ^31^.

Investigaciones realizadas en pandemia en Brasil encuentran estados donde primaron las decisiones basadas en intereses políticos y aquellos donde primaron decisiones basadas en criterios técnicos ^24^, identificando fallas en la coordinación-cooperación entre Estado Nacional y subnacionales ^32^. En Argentina las tensiones por cuestiones de federalismo y de autonomía provincial influyeron respecto al cierre de escuelas en pandemia, donde la disputa entre el Ejecutivo Nacional y Buenos Aires llegó a la Corte Suprema de Justicia ^33^. No se ha publicado sobre federalismo y políticas públicas sobre tecnologías sanitarias en pandemia.

Las decisiones sobre políticas públicas en contextos de incertidumbre son buenos casos de análisis al extremar algunas características de procesos de decisión presentes en contextos habituales. La crisis provocada por la pandemia no es solo excepción o ruptura, sino que hay mucha continuidad en ella, dado que las personas siguen apelando a sus marcos de referencia ^34^. Caduff ^35^ describe en las epidemias el rol de los “expertos”, que compiten para volverse influyentes. En esta pandemia se observaron disputas por “el saber” entre distintos “expertos”, que ocurrieron, tanto en artículos científicos ^36^, como en medios de comunicación y reuniones privadas con tomadores de decisión ^37^.

Este artículo propone analizar cómo se procesaron en la federación argentina los movimientos de coordinación vs. cooperación, y de autonomía vs. reparto de autoridad, explorando especialmente las asimetrías entre las diferentes entidades en la implementación de las políticas públicas durante la pandemia y las influencias de otros actores sobre las decisiones relativas al uso de tecnologías sanitarias.

## Métodos

Se realizó una triangulación de métodos cuali-cuantitativos, recolectando información del período 2020-2021. Se seleccionaron los casos del ibuprofeno inhalado, ivermectina, plasma de convalecientes y suero equino.

Se llevó a cabo una búsqueda bibliográfica y revisión documental, indagando sobre procesos y resultados de la toma de decisiones en los diferentes Ministerios de Salud. Se analizaron informes técnicos oficiales, leyes, fallos judiciales, noticias periodísticas y acciones de la sociedad civil en redes sociales e internet.

Se consultaron las recomendaciones técnicas oficiales de la OMS, de la Organización Panamericana de la Salud (OPS), de la CONETEC y de la RedARETS ^21^^,^^38^ que mostraron completa coherencia entre ellas, recomendando en contra de las tecnologías sanitarias seleccionadas, por lo que en adelante, se las denominará en forma genérica “recomendaciones de la OMS”, y las recomendaciones de cada Ministerio de Salud de las provincias argentinas. Las recomendaciones de las sociedades científicas más relevantes fueron recabadas a partir de sus páginas *web* oficiales. Se mapeó la cobertura y la recomendación de cada tecnología, y el partido político gobernante en cada provincia, con el objetivo de explorar la trama de los actores intervinientes.

Se obtuvo información a través de una encuesta a actores clave relacionados con la toma de decisión sobre tecnologías sanitarias en los Ministerios de Salud de la Nación y de las provincias argentinas. La construcción de la encuesta siguió los pasos secuenciales de generación de dominios e ítems con base en la revisión bibliográfica y discusión con expertos, reducción de ítems, evaluación de validez y confiabilidad, prueba piloto, realización de ajustes, difusión y análisis. Fue enviada a sujetos que reunían los criterios de inclusión, pertenecientes a todos los Ministerios de Salud de las provincias y de la Nación en 2020-2021. La encuesta consistió en un cuestionario en formato electrónico con consentimiento informado. Tuvo como objetivos reunir información sobre la conformación de los equipos asesores, la participación de influyentes internos y externos, los procesos y resultados sobre tecnologías sanitarias. Se utilizaron las respuestas para seleccionar sujetos a entrevistar en profundidad.

Se realizaron entrevistas semiestructuradas para explorar aspectos vivenciales, buscando casos que ilustren los procesos de toma de decisión en políticas públicas durante la pandemia. Las entrevistas se realizaron en forma presencial o por plataforma audiovisual, pidiendo autorización para su grabación y posterior análisis. Dos entrevistadores las realizaron, llevando un registro sobre impresiones, y lenguaje no verbal. Se realizaron 34 encuestas a tomadores de decisión y asesores de 14 Ministerios de Salud diferentes, y 8 entrevistas. Para la etapa analítica del presente artículo, nos basamos en el análisis de fuentes secundarias recopiladas en la investigación (informes técnicos, leyes, fallos judiciales, notas periodísticas) y fuentes primarias como las encuestas y entrevistas a integrantes de Ministerios de Salud de Argentina.

Sobre la base de los resultados, se planteó, dentro de la metodología de la investigación cualitativa, el abordaje de estudio de casos. Estos son “*un medio de organizar datos sociales, preservando el carácter unitario del objeto social estudiado*” ^39^ (p. 422). El estudio de caso se concentra en el conocimiento experiencial del caso y en la atención a la influencia de su contexto social y político ^40^, cuyo análisis puede iluminar el funcionamiento de un sistema social de un modo que una serie de afirmaciones políticas no pueden lograr ^41^. Fueron seleccionados como casos cuatro tecnologías sanitarias, explorando los diferentes contextos y procesos en cada Ministerio de Salud.

Se entendieron las políticas públicas como el conjunto de objetivos, decisiones y acciones que lleva a cabo un gobierno para solucionar los problemas que en un momento determinado los ciudadanos y el gobierno consideran prioritarios ^11^. La decisión de política pública (o *policy*) es aquella conformada por un acto voluntario, la existencia de alternativas, el proceso o secuencia de acciones y decisiones elementales que han acabado determinando su contenido y una potencial transformación o cambios en el mundo que nos rodea ^12^. La coordinación federativa o intergubernamental se entiende como formas de integración, intercambio y toma de decisiones conjunta presente en las federaciones ^42^.

Las tecnologías sanitarias son cualquier intervención que pueda ser utilizada en promoción de la salud, prevención, diagnóstico o tratamiento de una enfermedad, rehabilitación o cuidados prolongados ^43^. Adherimos a la concepción de que toda tecnología es política, ya que, lejos de ser neutral, refleja los planes, propósitos y valores de nuestra sociedad ^44^. La ETS es un proceso multidisciplinario que utiliza métodos explícitos para determinar el valor de una tecnología sanitaria en diferentes puntos de su ciclo vital. Su propósito es informar la toma de decisiones con el fin de promover un sistema de salud equitativo, eficiente y de calidad ^35^. El denominado “pensamiento crítico latinoamericano” pone en discusión el cientificismo, desacralizándolo y centrando la atención en los procesos sociales, culturales, políticos, económicos en relación con los conocimientos generados en ciencia y tecnología ^45^.

Este estudio fue evaluado por la Comisión de Bioética de la Provincia de Neuquén, Argentina (nota 87/2021).

## Resultados

En 2020 el partido político a cargo del Ejecutivo Nacional (Frente de Todos), con Alberto Fernández como presidente, contaba con gobernadores aliados en la mayoría de las provincias argentinas, siendo solo seis las provincias gobernadas por el partido opositor Juntos por el Cambio (coalición que había gobernado el Estado Nacional en el periodo inmediatamente anterior, con Mauricio Macri como presidente). En materia de política sanitaria, estos partidos gobernantes habían tenido fuertes contrastes: el gobierno de Frente de Todos se presentaba como una línea de continuidad con los gobiernos del Frente para la Victoria, liderado por Néstor Kirchner y Cristina Fernández; y al poco tiempo de haber asumido, restituyó el Ministerio de Salud, que había sido rebajado a la condición de “Secretaría” durante el gobierno presidido por Mauricio Macri. Este último había abogado por un discurso de recorte del gasto público, que impactó fuertemente en una disminución de las capacidades del Estado Nacional.

**Figura 1:**
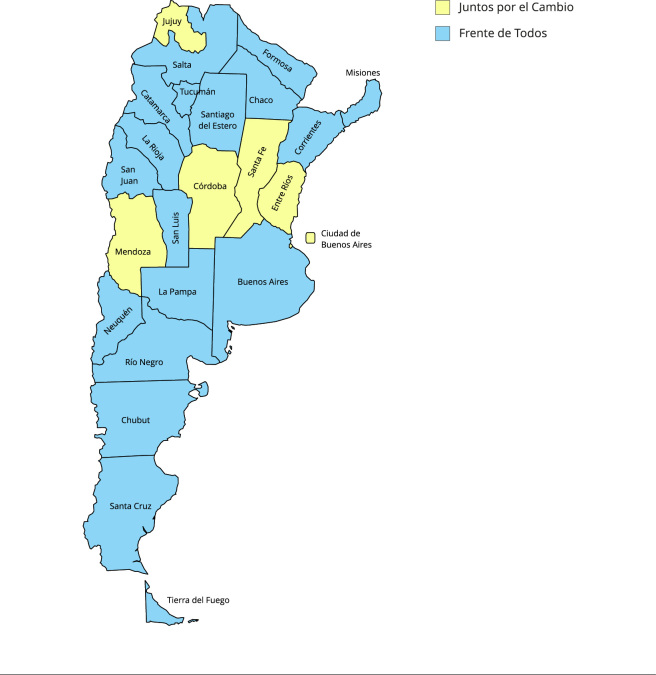
Partidos políticos provinciales. Argentina, 2020.

Si bien la clasificación binaria de la orientación político-partidaria en “oficialismo-oposición” puede ser una simplificación excesiva, en cuanto a que podría ocultar distintos niveles de colaboración-cooperación y alianzas entre las 24 provincias y el Estado Nacional, a los fines operativos permite evaluar su relación con la toma de decisiones sobre las tecnologías sanitarias, como fue realizado en otros estudios ^26^. Esto se observa en la Figura 1.

Las decisiones sobre tecnologías sanitarias fueron sumamente variables entre distintos Ministerios de Salud de Argentina, y contrarias a recomendaciones de la OMS. No se observa correlación entre el partido político gobernante provincial y la adherencia a recomendaciones técnicas.

Dentro del Ministerio de Salud de la Nación se observaron contradicciones y disputas por “el saber” entre áreas de la burocracia ministerial. Si bien este Ministerio de Salud adhirió a muchas recomendaciones técnicas de la OMS y de sus propias áreas técnicas (CONETEC), no fue así en el caso del suero equino y del plasma de convaleciente ^21^^,^^46^^,^^47^^,^^48^.

La sanción de una ley nacional de uso de plasma en 2020 puede ser interpretada como una presión externa desde el Poder Legislativo, donde el oficialismo no contaba con una mayoría automática. El Ministerio de Salud de la Nación, asesorado por diversas sociedades científicas, luego emitió una resolución recomendando el uso de esta tecnología sanitaria ^48^. Los Ministerios de Salud de todas las provincias adhirieron, lo que implicó el uso masivo de plasma en Argentina, distrayendo importantes recursos y exponiendo a la población a potenciales efectos adversos, por fuera del contexto de investigaciones científicas.

Por el suero equino no se sancionaron leyes. Pese a las recomendaciones negativas de la OMS, de la CONETEC, de la OPS y de la RedARETS, fue recomendada por el Ministerio de Salud de la Nación ^47^, y contó con una resolución de la Dirección Nacional de Calidad ^48^. Diversas sociedades científicas asesoraron al Ministerio de Salud de la Nación en la elaboración de esta norma, dictaron webinarios y conferencias de prensa promoviendo su uso ^49^. Se estimaba que 221 instituciones médicas del país habían administrado unas 25.000 dosis de suero equino ^50^; equivalente a un gasto de 18,6 millones de dólares. El 90% de los Ministerios de Salud compraron y recomendaron suero equino. No se identificaron acciones judiciales por acceso al suero equino. Este fármaco es producido por un laboratorio argentino, cuya influencia a través de voceros e investigadores en medios de comunicación y campañas de marketing fue importante y efectiva. Al finalizar un estudio multicéntrico, pese a que los resultados no demostraron eficacia ^21^, se lo anunció en medios de prensa como un éxito rotundo ^51^. Inmediatamente, fue seguido por un estudio de farmacovigilancia en diversos centros del país, lo que parece más un estudio de siembra, cuyo objetivo principal es promocionar el producto ^52^.

La alineación política partidaria provincial no parece haber influido en ninguno de los dos casos, ya que tanto Ministerios de Salud de provincias oficialistas como opositoras compraron suero equino y elaboraron y recomendaron plasma de convaleciente.

El caso del ibuprofeno inhalado en Argentina revistió características diferentes a estas dos tecnologías sanitarias analizadas. Su recomendación surgió a partir del liderazgo de investigadores provinciales, que lo estudiaban para otras enfermedades respiratorias y propusieron utilizarlo para COVID-19. Rápidamente, se sumaron apoyos en ámbitos académicos provinciales y de un importante número de farmacéuticos magistrales que lo elaboraron en forma gratuita en todo el país. Estos conformaron una red que se mantuvo muy activa en medios y redes sociales, brindó capacitaciones y ofreció el ibuprofeno inhalado solidariamente entre distintas provincias ^53^. Organizaciones no gubernamentales (ONG) provinciales elaboraron proyectos, presionaron y lograron la sanción de leyes provinciales autorizando su uso, aun cuando la OMS no había recomendado su utilización. El Poder Judicial de diversas provincias intervino en casos individuales, obligando su utilización, aun en contra de la opinión de los equipos médicos tratantes ^54^. En algunas provincias, el Poder Judicial obligó al Ministerio de Salud a revisar su posición en contra del ibuprofeno inhalado ^55^. En la Tabla 1 se resumen las coberturas, recomendaciones y apoyos en relación con las cuatro tecnologías sanitarias mencionadas.

**Tabla 1:** Cuadro comparativo de medidas, apoyos y recomendaciones para ivermectina, ibuprofeno inhalado, plasma de convaleciente y suero equino para COVID-19 en Argentina.

Medidas, apoyos y recomendaciones	Ivermectina	Ibuprofeno inhalado	Plasma de convaleciente	Suero equino
Recomendación de OMS/OPS	No utilizar	No utilizar	No utilizar	No utilizar
Recomendación del Ejecutivo Nacional	No utilizar	No utilizar	Utilizar *	Contradictoria **
Porcentaje de Ministerios de Salud que lo recomendaron y/o cubrieron en Argentina	50%	77%	100%	94%
Sancion de leyes apoyando su uso	Sí, provinciales	Sí, provinciales	Sí, ley nacional	No
Recursos judiciales obligando su uso	Sí, colectivos	Sí, colectivos e individuales	No	Sí, individuales
Apoyos	Investigadores, ONG, prensa	Investigadores, ONG, prensa	Investigadores, sociedades científicas, prensa	Investigadores, sociedades científicas, industria farmacéutica, prensa

OMS: Organización Mundial de la Salud; ONG: organizaciones no gubernamentales; OPS: Organización Panamericana de la Salud.

* Se sancionó una ley nacional de uso de plasma de convaleciente, y luego el Ejecutivo Nacional publicó una resolución desde el Ministerio de Salud de la Nación recomendando su uso;

** La Comisión Nacional de Evaluación de Tecnologías Sanitarias no recomendó su utilización, mientras que la Dirección Nacional de Calidad publicó una resolución recomendando su uso y el ministro de Salud de la Nación recomendó su utilización en acta de reunión con ministros de Salud de todo el país en el marco del Consejo Federal de Salud.

No se cuenta con estadísticas oficiales para conocer su verdadera utilización, ni la incidencia de eventos adversos. La alineación política provincial no parece correlacionarse con su utilización, pero se identificó el caso de una provincia donde esto parece haber tenido una fuerte influencia. En la Provincia de Santa Cruz, alineada históricamente con el oficialismo a cargo del Ejecutivo Nacional, un colectivo de la sociedad civil presionó por la sanción de una ley por ibuprofeno inhalado ^56^. Ante la negativa para sancionar la ley, la presión ejercida desde redes sociales y medios periodísticos fue in crescendo, alcanzando niveles de agresión muy elevados y presentándola como una discusión política entre oposición y oficialismo. Portadas de medios digitales contaban el número de muertos, y se los atribuían personalmente a una legisladora oficialista con nombre y apellido, identificándola como la responsable por oponerse a la ley ^57^. Esta utilización de los medios de comunicación excedió lo visto en otros debates por tecnologías sanitarias durante la pandemia. A nivel nacional se vieron agresiones similares en la discusión por la compra de vacunas Sputnik y en el debate por el cierre de escuelas, que llegaron a judicializar sus demandas ^33^^,^^58^.

El caso de la ivermectina en Argentina tuvo particularidades que lo diferencian de otras tecnologías sanitarias mencionadas. En muchas provincias la solicitud de su utilización comenzó en miembros del equipo de salud, haciéndose eco de investigaciones nacionales ^59^. Aproximadamente la mitad de los Ministerios de Salud e Argentina la recomendaron. La sociedad civil se organizó desplegando una amplia gama de recursos para peticionar por acceso a ivermectina. Se realizaron peticiones al Ministerio de Salud, al Defensor del Pueblo, proyectos de ley, y reclamos judiciales. En algunas provincias, los colectivos que reclamaron por el acceso a la ivermectina coincidieron en el reclamo por acceso al ibuprofeno inhalado, en contra de las vacunas para COVID-19, en contra del uso de barbijos y llevarían adelante acciones violentas contra funcionarios del Ministerio de Salud provincial ^60^. Pero no en todas las provincias fue igual. En la Provincia de Neuquén, ONG peticionaron al Defensor del Pueblo, quien solicitó una mediación con el Ministerio de Salud por la ivermectina. Cinco Ministerios de Salud provinciales implementaron protocolos de utilización profiláctica de ivermectina para personal de salud ^16^. Dos laboratorios de producción pública de medicamentos detuvieron otras líneas de producción para volcarse a la fabricación de ivermectina en gran escala ^51^^,^^61^. No se observó una alineación político-partidaria de los Ministerios de Salud que recomendaron y utilizaron ivermectina en contra de las recomendaciones del Ministerio de Salud de la Nación y de la OMS.

## Discusión

Los casos analizados muestran heterogeneidad en cuanto a actores, roles y trayectorias de las políticas públicas en pandemia. Mientras que la ley por plasma de convaleciente surgió como iniciativa desde el Congreso Nacional argentino, el suero equino fue promocionado por el laboratorio productor y el Ministerio de Salud de la Nación, y las iniciativas por ibuprofeno inhalado e ivermectina surgieron inicial y principalmente desde la sociedad civil e investigadores locales. Los proyectos de ley por acceso a ibuprofeno inhalado e ivermectina eran idénticas en su redacción entre provincias, lo que da cuenta de una articulación interprovincial entre ONG e investigadores. Se identificaron fallos judiciales individuales y colectivos por estos dos fármacos, que obligaron a administrarlos, pese a que los Ministerios de Salud los habían rechazado en línea con los informes técnicos de la OMS, de la CONETEC y de la RedARETS ^21^^,^^38^.

Las tensiones por cuestiones de federalismo y autonomías provinciales no surgieron de parte de los encuestados y entrevistados cuando se indagó sobre estas tecnologías sanitarias, incluso en aquellas provincias gobernadas por fuerzas políticas opositoras al partido a cargo del Ejecutivo Nacional ^62^. Esto pone de manifiesto que las tensiones relacionadas con el balance entre intereses político-partidarios y federalismo-autonomía, si bien pueden haber jugado algún papel en casos puntuales, no parecen explicar la heterogeneidad observada. Las asimetrías entre el papel de coordinación de la entidad nacional y la autonomía de las entidades parecen relacionarse con una trama compleja de actores que entran en tensión, acuerdan, participan de discusiones y conflictos, que terminan siendo también influyentes de la política pública local.

En uno de los casos, los embates de parte de la oposición hacia políticos del partido gobernante provincial se relacionaron con el debate público por el acceso al ibuprofeno inhalado ^57^. Respecto a otras tecnologías sanitarias para COVID-19 como las vacunas, el presidente y el ministro de Salud de la Nación fueron denunciados penalmente por “envenenamiento” por parte de legisladores del partido opositor ^58^. En provincias gobernadas por fuerzas políticas alineadas con el gobierno nacional también se tomaron decisiones sobre tecnologías sanitarias contrarias a sus recomendaciones. La política partidaria no parece poder explicar completamente las decisiones tomadas sobre cobertura de tecnologías sanitarias en pandemia en Argentina.

Hacia adentro de los mismos Ministerios de Salud se objetivaron contradicciones y disputas por “el saber”. Se identificaron investigadores y profesionales de la salud que disputaron el conocimiento y operaron en muchos casos buscando influir desde medios de comunicación y de ONG ^63^.

Todos los Ministerios de Salud percibieron una fuerte presión por parte de los medios de comunicación, y en algunos casos enfrentaron campañas en redes sociales presionando por la cobertura de tecnologías sanitarias. El rol de los medios de comunicación y redes sociales en esta pandemia merece ser destacado. Por un lado, transmitieron información, en muchos casos valiosa, pero también generaron en muchos casos ansiedad, desinformación a gran escala, infodemia, y fueron utilizados como elemento de presión sobre los tomadores de decisión ^64^. El consumo acrítico de redes sociales demostró influir en el uso de tecnologías sanitarias como las vacunas ^65^. Los mensajes en Twitter de famosos, generaron millones de interacciones en redes sociales y se vieron asociados a un mayor consumo de cloroquina ^66^. Estos elementos no tenían la misma trascendencia cuando los modelos de toma de decisión clásicos fueron elaborados, pero deben ser considerados actualmente.

Observamos una fuerte movilización de recursos desde ONG y colectivos sociales que se agruparon presionando sobre los tomadores de decisión por el acceso a tecnologías sanitarias. Esto fue más importante para bregar por el acceso a ibuprofeno inhalado e ivermectina, coexistiendo, y haciendo sinergia en muchos casos, con agrupaciones pseudocientíficas, grupos antivacunas, pero también con investigadores universitarios.

La ivermectina, el ibuprofeno inhalado y el plasma de convaleciente no contaban con patente, se podían producir a nivel nacional y a costos relativamente bajos; fueron promocionados por líderes de opinión locales, apoyados por ONG, y medios de comunicación. El ibuprofeno inhalado y la ivermectina no contaron con apoyo de sociedades científicas reconocidas, mientras que el plasma de convaleciente contó con apoyo de la Sociedad Argentina de Infectología y diferentes sociedades relacionadas con la hemoterapia y la medicina transfusional, y el suero equino contó con apoyo explícito de las sociedades argentinas de Infectología, Terapia Intensiva y Medicina. Probablemente, para las cuatro tecnologías sanitarias, existió en parte de la comunidad científica, política y en el equipo de salud, cierto “orgullo” por tratarse de fármacos o procedimientos desarrollados en nuestro país.

El caso de suero equino en Argentina se diferencia de los otros tres casos, y muestra la influencia del laboratorio productor, mediante la utilización de estrategias de marketing frecuentes de la industria farmacéutica ^52^.

Se observa una débil coordinación por parte del Ministerio de Salud de la Nación en lo que respecta al uso racional de ciertas tecnologías sanitarias para la pandemia. Esto no ocurrió con las vacunas para COVID-19, ni con las medidas de distanciamiento social que fueron más homogéneas a lo largo de toda la federación. En cuanto al plasma, al suero equino, al ibuprofeno inhalado y a la ivermectina, se observa la influencia de varios otros actores para que fuera recomendada en contra de la evidencia científica. Esto deja de manifiesto fallas en la coordinación-cooperación en pandemia que también fueron descriptas en Brasil ^24^^,^^32^ y deben ser analizadas para el afrontamiento de presentes y futuros desafíos.

## Conclusiones

El caso de las tecnologías sanitarias seleccionadas en los Ministerios de Salud de Argentina permite observar un comportamiento no secuencial de las políticas sobre medicamentos para COVID-19, que parece contradecir a algunos de los acercamientos más normativos y racionalistas que ven las políticas como modelos lineales y pulcros de toma y ejecución de decisiones. La realidad muestra cuán desordenadas, complejas y no lineales pueden ser las políticas públicas, y desacredita la suposición de que las políticas necesariamente tienen un autor o arquitecto coherente. Muchas veces la mejor manera de analizar las políticas es en cuanto a los efectos y no tanto a respecto de los orígenes o causas, ya que las políticas tienen agencia, independientemente de la voluntad de sus creadores ^12^. A diferencia de lo registrado en otros países, como es el caso de Brasil respecto a las medidas de distanciamiento social entre sus Estados, las cuestiones político-partidarias no parecen poder explicar completamente la heterogeneidad en las decisiones tomadas en distintas provincias argentinas en pandemia.

Como eje central, se identifica la heterogeneidad y la complejidad como característica distintiva de la toma de decisiones sanitarias en pandemia. Esto se observó entre los Ministerios de Salud, y hacia adentro de los mismos a lo largo de la pandemia y según diferentes tecnologías sanitarias. La influencia de actores internos y externos a la administración fue también heterogénea y la trama de interacciones compleja. Si bien puede argumentarse que el Estado Nacional Argentino respetó la autonomía subnacional para tomar decisiones sobre tecnologías sanitarias en pandemia, cabe preguntarse en qué medida esto fortalece o debilita la federación; especialmente ante aquellos casos donde las provincias utilizaron en su población tecnologías sanitarias inadecuadas sin fundamentos científicos de eficacia y seguridad.

En los países federales, donde las políticas públicas nacionales son compartidas entre esferas de gobierno, la cooperación y no la coordinación sería el concepto más apropiado para abordar este reparto, debido a la autonomía constitucional de las entidades federales ^67^. Distintos autores como Arretche ^68^ o Bednar ^69^ plantean que ni la cooperación ni la coordinación se dan en forma natural, y lo que mejor parece funcionar son los incentivos que hacen racional la adhesión de los participantes en las políticas y sus agencias. Un estímulo para adherir a las políticas públicas vinculadas al uso racional de tecnologías sanitarias en pandemia podría haber consistido en facilitar el acceso a precio subsidiado o gratuito para las provincias de aquellas tecnologías sanitarias cuyo uso se quería incentivar, y gravar o dificultar el acceso (a través de las importaciones, impidiendo su producción en laboratorios públicos, etc.) de aquellas tecnologías sanitarias cuyo uso se deseaba desalentar. Resulta central lograr dividir la autoridad entre los estados y el gobierno federal para que la rivalidad no destruya el potencial de la unión, alcanzando un equilibrio entre autonomía y reparto de autoridad, sin dejar de proteger y garantizar el derecho a la salud de toda la población.
